# Reduced phenotypic plasticity evolves in less predictable environments

**DOI:** 10.1111/ele.13598

**Published:** 2020-08-31

**Authors:** Christelle Leung, Marie Rescan, Daphné Grulois, Luis‐Miguel Chevin

**Affiliations:** ^1^ CEFE Université de Montpellier CNRS EPHE IRD Université Paul Valéry Montpellier 3 Montpellier France

**Keywords:** *Dunaliella salina*, environmental stochasticity, experimental evolution, fluctuating environment, Phenotypic plasticity, predictability

## Abstract

Phenotypic plasticity is a prominent mechanism for coping with variable environments, and a key determinant of extinction risk. Evolutionary theory predicts that phenotypic plasticity should evolve to lower levels in environments that fluctuate less predictably, because they induce mismatches between plastic responses and selective pressures. However, this prediction is difficult to test in nature, where environmental predictability is not controlled. Here, we exposed 32 lines of the halotolerant microalga *Dunaliella salina* to ecologically realistic, randomly fluctuating salinity, with varying levels of predictability, for 500 generations. We found that morphological plasticity evolved to lower degrees in lines that experienced less predictable environments. Evolution of plasticity mostly concerned phases with slow population growth, rather than the exponential phase where microbes are typically phenotyped. This study underlines that long‐term experiments with complex patterns of environmental change are needed to test theories about population responses to altered environmental predictability, as currently observed under climate change.

## Graphical abstract

Many organisms can modify their phenotypes in response to the environment in which they develop, through phenotypic plasticity. However, such plastic responses may not necessarily be adaptive, depending on how the produced phenotype matches selective pressures across environments. Theory predicts that the degree of plasticity should evolve in response to the predictability of environmental fluctuations through time and space, but this is extremely difficult to demonstrate in the wild. Here, we have used long‐term experiments with controlled patterns of environmental fluctuations, to show unambiguously that lower phenotypic plasticity evolves in environments that fluctuate less predictably. This has important consequences for the fate of populations in response to climate change and other environmental disturbances.

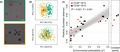

## INTRODUCTION

Phenotypic plasticity, the ability of a given genotype to produce alternative phenotypes depending on its environment of development or expression, is a major mechanism for responding to environmental variation across the tree of life (Scheiner, 1993; Schlichting and Pigliucci, 1998; West‐Eberhard, 2003). In recent years, the study of phenotypic plasticity has gained prominence in evolutionary ecology, with the realisation that it contributes a substantial part to observed phenotypic change in the wild, notably in response to climate change (Gienapp *et al*., 2008; Merilä and Hendry, 2014). Because phenotypic change is generally a strong determinant of population dynamics (Pelletier *et al*., 2007; Ozgul *et al*., 2010; Ellner *et al*., 2011), this implies that phenotypic plasticity can strongly impact population growth and extinction risk in a rapidly changing world, to an extent that depends on the rate and pattern of environmental change (Chevin *et al*., 2010; Reed *et al*., 2010; Vedder *et al*., 2013; Chevin *et al*., 2013b; Ashander *et al*., 2016; Phillimore *et al*., 2016).

However, despite the ubiquity and ecological importance of phenotypic plasticity, proving its adaptiveness for any particular trait and organism is particularly challenging (Ghalambor *et al*., 2007). Most evidence that plasticity is adaptive is instead indirect, for instance through comparison of the direction of plastic vs. evolved responses to a novel environment (Ghalambor *et al*., 2015), except for rare studies where plastic responses have been genetically engineered to compare the fitness of plastic vs. non‐plastic genotypes across environments (Dudley and Schmitt, 1996). However, beyond the putative advantage of being plastic, a more meaningful and quantitative question is whether a given degree of plasticity is adaptive. This question was thoroughly addressed theoretically, and the predictability of environmental variation was identified as a key determinant of the adaptiveness of plasticity, and driver of its long‐term evolution (Gavrilets and Scheiner, 1993; de Jong, 1999; Lande, 2009; Reed *et al*., 2010; Botero *et al*., 2015; Tufto, 2015). In particular, high degree of plasticity is expected to evolve in highly predictable environments, whereas reduced phenotypic plasticity should evolve in environments that fluctuate less predictably, because this leads to plastic responses that do not match future selective pressures (Gavrilets and Scheiner, 1993; de Jong, 1999; Lande, 2009; Botero *et al*., 2015; Tufto, 2015). Lower plasticity may also evolve in a constant environment, if there are costs associated to the maintenance or production of plasticity (DeWitt *et al*., 1998). Nevertheless, these predictions still largely lack direct empirical evidence (but see Scheiner and Yampolsky, 1998; Dey *et al*., 2016), owing to the difficulty in manipulating the variability and predictability of the environment over evolutionary times. In addition, multiple independent replicates of environmental fluctuations are needed in order to account for their inherent randomness (environmental stochasticity), but this is difficult to achieve in nature.

A useful alternative to circumvent these limitations is to perform long‐term laboratory experiments under controlled, yet ecologically realistic, patterns of environmental fluctuations. This approach, which was previously advocated by Chevin *et al*. (2013a), allows controlling for the level of environmental predictability, with all other things being equal – including the mean and variance of the environment – and also permits sufficiently large duration and replication to unambiguously observe evolutionary responses to stochastic environments. Here we have applied this approach with the unicellular microalga *Dunaliella salina*, the main primary producer in hypersaline environments such as continental salt lakes, coastal lagoons and salterns (Oren, 2005; Ben‐Amotz *et al*., 2009). The cell shape and content of this microalgae are known to respond plastically to salinity over different timescales, allowing for both immediate morphological responses to sudden osmotic stress, and slower physiological adjustments involving the production of metabolites (including glycerol and carotene) inside the cell (Oren, 2005; Ben‐Amotz *et al*., 2009). Several of these traits can be measured at the individual level and at high‐throughput, pushing work on phenotypic plasticity towards the realm of phenomics (Houle *et al*., 2010; Pendergrass *et al*., 2013; Yvert *et al*., 2013). Experimental evolution has been successfully performed previously with the closely related species *Dunaliella tertiolecta* (Malerba *et al*., 2018). Furthermore, we have recently shown that phenotypic plasticity plays a crucial role in the population dynamics and extinction risk of *Dunaliella salina* in a randomly fluctuating environment (Rescan *et al*., 2020). This makes *D*.* salina* particularly well‐suited to investigate experimentally the evolution of phenotypic plasticity in response to environmental predictability.

## MATERIAL AND METHODS

### Experimental evolution

**Figure 1:**
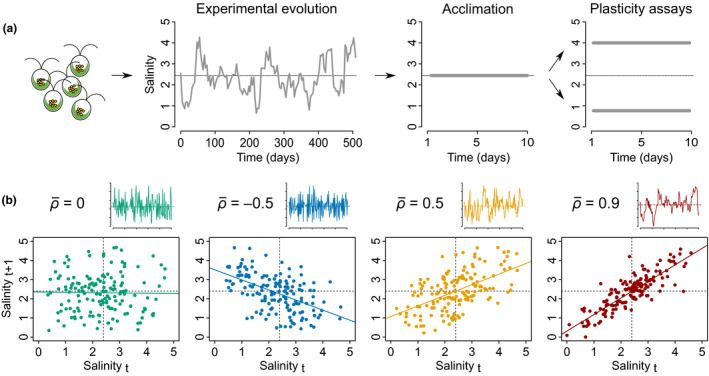
Long‐term evolution experiment under variable environmental predictability. (a) The different steps of the experiment are illustrated. (b) Examples of the four categories of expected long‐term temporal autocorrelation ($\bar{\rho }$). For each category, the small graph represents the associated time series for a given population (same axes as in Figure 1a), with mean salinity shown as horizontal dotted line. The larger graphs represent the relationship between subsequent salinities in these time series. Note that the expected autocorrelations$\bar{\rho }$ = −0.5 and $\bar{\rho }$ = 0.5 lead to different magnitudes of salinity changes, but display the same predictability of environmental changes ($\bar{\rho }$ ^2^ = 0.25).

We followed up on the experimental evolution protocol initiated by Rescan *et al*. (2020), which we pursued for several hundred generations. Briefly, we used two genetically related strains (CCAP 19/12 and CCAP 19/15) of the halophilic unicellular microalga *Dunaliella salina,* which can tolerate a broad range of salinities. Long‐term experimental evolution occurred from August 2017 to January 2019, during which we exposed 32 populations to randomly fluctuating salinity, and four populations to a constant intermediate salinity ([NaCl] = 2.4 M). Salinity changes occurred twice a week, by 20% dilution into 800 µl of fresh medium to avoid population extinction (Rescan *et al*., 2020), using a liquid‐handling robot (Biomek NXP Span‐8; Beckman Coulter). At each transfer, the target salinity was achieved by mixing the required volumes of hypo‐ ([NaCl] = 0 M) and hyper‐ ([NaCl] = 4.8 M) saline media, after accounting for dilution of the pre‐transfer salinity (Rescan *et al*., 2020). Salinity then remained constant for the next three or four days until the next transfer, during which individual cells could adjust their phenotype in response to the new salinity, as well as reproduce both clonally and sexually. We attained at least 139 salinity transfers and more than 500 generations (assuming ~ 1 generation per day (Ben‐Amotz *et al*., 2009)). The populations in fluctuating environments were subjected to independent random time series over a continuous range (first‐order autoregressive process, AR1), rather than a more artificial alternation of low vs. high salinity treatments. All the lines had the same long‐term stationary mean (µ = 2.4 M [NaCl]) and variance (σ = 1) of salinity, but they differed in how salinity at a given time depends on the previous salinity, prior the last transfer, as determined by the temporal autocorrelation ρ of salinity (Fig. 1a and b) (Rescan *et al*., 2020). The predictability of environmental change upon these transfers depended on ρ^2^, the proportion of the temporal variance in salinity explained by the previous salinity (Fig. 1b). There were four autocorrelation treatments, for which the expected autocorrelations (over infinite time) were $\bar{\rho }$ = −0.5, 0, 0.5 and 0.9 (Fig. 1; see Rescan *et al*. (2020) for detailed protocol), but the realised autocorrelation over the duration of the experiment spanned a continuum of values. Note that even high predictability treatments (with large values of ρ^2^) are still stochastic (random), and therefore differ from deterministic, periodic cycles. All populations were cultivated in 1.1ml of 96‐deepwell plates (Axygen®; Corning Life Sciences) in an artificial seawater prepared as described in Rescan *et al*. (2020), at constant temperature (24°C) and 12/12 lighting (200 µmol.m^−2^.s^−1^).

At the end of the experiment, we genetically confirmed that strains were not cross‐contaminated after *c*. 1.5 year of experiment. Specifically, for each evolved line, we amplified one mitochondrial locus (333 pb fragment using the primers DsMt1‐For [5’‐GGTTAGTCATAGTTGGAGGT‐3’] and DsMt1‐Rev [5’‐GAAAACCTAACATGGCTAAGC‐3’] and one chloroplast locus (372 bp fragment using the primers DsChl1‐For [5’‐TTTAGGCGAATCCATAAGAG‐3’] and DsChl1‐Rev [5’‐CCAAGCAGGTGAATTAGCTTTG‐3’]), specific to CCAP 19/12 and CCAP 19/15 respectively.

### Phenotypic plasticity assays

To assess the degree of plasticity of the 36 evolved lines, we compared individual cell morphologies between two salinities near the extremes of their historical range ([NaCl] = 0.8 M and 4.0 M). These lines started with potentially genetically diverse strains, and were certainly also polymorphic at the end of experimental evolution. To verify that morphological changes between salinities over the 10‐day assay were due to plastic responses rather than environment‐specific selection, we also performed the phenotypic plasticity assays with 20 additional isogenic populations derived from five heterogenic experimental lines. One of these experimental lines was the CCAP 19/12 strain that evolved in constant salinity (2.4 M) and four others were from the CCAP 19/15 strain that evolved in constant (2.4 M) or fluctuating (with targeted autocorrelation ρ = −0.5, ρ = 0 and ρ = 0.9) salinities. For each of these five experimental populations, we founded four different populations from single cells isolated using cells‐sorting flow cytometry (BD *FACSAria*™ IIu; Biosciences‐US). Because *Dunaliella salina* is haploid, we expected all cells to be genetically identical in populations founded from a single one.

The 36 lines were sampled from different salinities and at different population sizes at the end of the evolutionary experiment (Rescan *et al*., 2020). To ensure that all cells were in similar physiological states and at similar population densities at the beginning of the phenotypic plasticity assay, we first acclimated them for 10 days in the same environmental conditions. We transferred 400 µl of each experimental population into 50 ml flasks (CellStar®; Greiner BioOne) with 25 ml of fresh medium at salinity [NaCl] = 2.4 M, temperature 24°C and light intensity 200 μmol m^−2^ s^−1^ for 12:12 h light/dark cycles.

Following the acclimation step, we inoculated *c*. 2 × 10^4^ cells.ml^−1^ of each population into low (0.8 M) or high (4.0 M) salinity medium (Fig. 1), to track their population density and morphological traits for 10 days. For 24 populations (including 14 randomly chosen evolved lines and 10 isogenic populations), we also inoculated the same number of cells into 2.4 M salinity medium to assess morphological changes independent from salinity changes. We randomly placed all conditions in 2 ml 96‐deepwell plates at 24°C and 12/12 lighting with 200 μmol m^‐2^ s^‐1^ light intensity.

When placed in a new salinity, *D. salina* undergoes changes in cell shape and content, reflecting an osmotic adaptability that unfolds over different timescales (Ben‐Amotz *et al*., 2009). Rapid changes in cell volume in response to changes in extracellular osmolarity are made possible by lack of a rigid cell wall. This then triggers longer physiological responses involving the production of osmolites (especially glycerol), changes in gene expression and the production of diverse salt‐induced proteins (Azachi *et al*., 2002; Oren, 2005; Ben‐Amotz *et al*., 2009), which all modify the cell content. To assess the changes in cell morphology, we passed a 150 µL sample of each population through flow cytometry at 11 time points: at the end of the acclimation step (day = 0), 4 h after environmental changes (day = 1) and once per day from day = 2 to day = 10.

Intrinsic structural parameters of cells were measured using a Guava^®^ EasyCyte™ HT cytometer (Luminex Corporation, TX, USA) with a laser emitting at 488 nm. The cytometer was calibrated each day of the experiment using the Guava^®^ EasyCheck™ kit, and settings were adjusted before each measurement. The flow rate was set to 1.18 µl.s^−1^, for 30s or until the number of counted events reached 5000. Data processing was carried out using Guava^®^ InCyte Software version 3.3, from which we exported list‐mode data files of measurements on the logarithmic scale. Non‐algal particles and dead algae were excluded from the analysis according a cytogram of emissions at Red‐B (695/50 nm) and Yellow‐B (583/26 nm) band pass filters, enabling a clear discrimination between algae populations and other events thanks to chlorophyll auto‐fluorescence (Rescan *et al*., 2020). We also discriminated doublets (i.e. single events that actually consists of two independent cells) from singlets according to the width of the electronic pulse measurement (FSC‐W) (Wersto *et al*., 2001). For events categorised as single alive *D. salina* cells, we specifically assessed the environment‐specific cell morphology using the Forward Scatter (FSC), Side Scatter (SSC) and fluorescence emission at 695/50 nm band pass filter (Red‐B) values as proxy for cells size, complexity (granularity, cytoplasmic contents) (Adan *et al*., 2017) and chlorophyll production (Papageorgiou, 2004) respectively. The density of the medium is likely to alter the light signal. To control for this effect, we subtracted from each individual measurement of FSC, SSC and Red‐B the mean value of the same parameter values among 1000 Guava^®^ EasyCheck™ calibrating beads placed in artificial seawater at the same salinity ([NaCl] = 0.8 M, 2.4 M or 4.0 M). We confirmed that traits we measured using flow cytometry closely matched cell size, shape and cellular contents including chlorophyll production, as there was 90.06% (*P* < 0.001) correspondence between PCAs performed with data from flow cytometry vs. image processing of epifluorescence microscopy (Procrustes analysis; Material S1 and Fig. S1). Finally, for each day and salinity, we determined population densities from the ratio of the count of events identified as alive *D*.* salina* cells to the total volume of acquisition.

### Statistical analyses

#### Population dynamics

For each population and salinity, we calculated the *per‐capita* growth rate of the population per day during the phenotypic plasticity assay, as $r=\frac{N\left(t\right)‐N\left(t‐1\right)}{N\left(t‐1\right)}$, where *N* is the population density (cells × ml^−1^), and *t* a time point of measurement (in days). For *N*(0), we used the initial density of 2 × 10^4^ cells.ml^‐1^ that we inoculated in each environment following the acclimation step. We describe days when *r* is highest as the exponential growth phase, whereas slower positive growth rates before and after the exponential phase are described as lag and stationary phases respectively.

#### Morphological variation and dynamics

Flow cytometry allows analysing thousands of individual cells in seconds, achieving high‐throughput phenotyping. Here, the low population density following transfer to a new environment (Days 1–2) limited the number of cells that could be measured by the flow cytometer, accounting for our measurement parameters. To keep a balanced design, we therefore randomly selected up to 150 events categorised as alive *D*.* salina* per conditions – that is population, environment and day –, totalizing more than 2 × 10^5^ individual cells for this study.

We then used a multivariate approach based on Redundancy Analyses (RDA) (Borcard *et al*., 1992) to assess the proportion of morphological variation explained by different predictors. For each *D*.* salina* cell, we used the morphological measurements (FSC, SSC and Red‐B cytometer values) as the multivariate response variable, and ancestral strain identity (CCAP 19/12 or CCAP 19/15), salinity during the plasticity assay, time point of measurement (day) and associated *per‐capita* growth rate, as explanatory variables. Variation partitioning was performed separately for populations that evolved in fluctuating vs. constant environments. For populations that evolved in fluctuating environments, the predictability of environmental change during long‐term evolution was included as an additional explanatory variable. As mentioned earlier, different time series within an autocorrelation treatment may vary in their realised autocorrelation, because of the randomness of the stochastic process in finite time. We therefore computed the realised environmental autocorrelation *ρ* as the correlation between salinities at two subsequent transfers, throughout each salinity time series. We then assessed the environmental predictability as ρ^2^, and used it as a continuous explanatory variable.

We quantified the effects of all explanatory factors or variables and their interactions through their contributions to total variation using a multivariate version of *R* ^2^, and tested the significance of each *R* ^2^ by ANOVA‐like permutation tests, using 999 randomisations of the data (Borcard *et al*., 1992; Legendre and Legendre, 1998; Peres‐Neto *et al*., 2006). A significant *salinity* × *predictability* interaction characterised the evolution of plasticity in response to our predictability treatment, a significant *day* × *salinity* interaction characterised a salinity‐specific ontogenic trajectory of morphology, and a significant three‐ways *day* × *salinity* × *predictability* interaction indicated that the evolution of plasticity had different magnitudes at different days along the ontogenic trajectory.

To illustrate the temporal changes in cells morphology following osmotic stress, we represented the mean cell morphology for each day in a morphospace for each targeted autocorrelation ($\bar{\rho }$). To do so, we first performed a Principal Component Analysis (PCA) of the morphological measurements of the entire data set, and calculated the centroid for each conditions (i.e. for a given salinity, day and population). We then represented the mean morphology with their standard errors, per day and salinity, by averaging over all populations for each targeted $\bar{\rho }$, and represented them along the two first PCA axes (Fig. 3b).

#### Evolution of the degree of plasticity

To assess how the magnitude of phenotypic plasticity evolved in our experiment, we first computed the Euclidian distance between the multivariate means calculated from the raw data of each experimental population measured at high (4 M) vs. low (0.8 M) salinity. We then compared this degree of plasticity among the experimental populations, to test whether it evolved according to environmental predictability. Specifically, for each day following transfer to the new salinity, we regressed the Euclidian distance of plastic change against ρ^2^, as index for environmental predictability. The significance of this regression was assessed by hierarchical nonparametric bootstrapping. We first resampled with replacement 32 populations among the 32 populations that evolved in fluctuating environments. For each population in each salinity, we resampled *n* = 150 cells with replacement. We then recomputed the degrees of plasticity of all populations, and regressed them against ρ^2^, iterating the full process 1,000 times. The proportion of simulations with slopes larger than 0 was used to assess the significance of whether lower plasticity evolved in populations that experienced less predictable environments.

We performed all statistical analyses in the environment R version 3.5.3 (R Core Team, 2019) with the vegan package version 2.5‐4 (Oksanen et al. 2019) for the multivariate analyses.

## RESULTS

### Evolution of reduced plasticity

**Figure 2:**
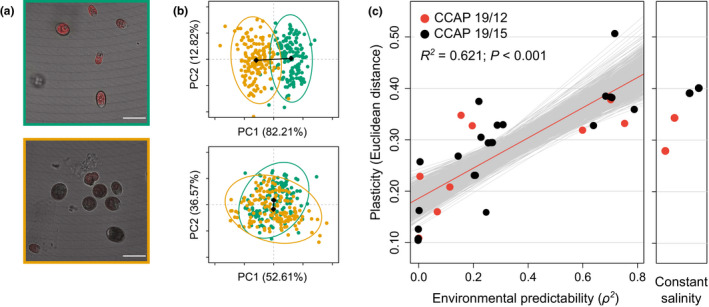
Evolution of morphological plasticity in response to environmental predictability. (a) Example of salinity‐specific cell morphologies. Red zones in composite images represent chlorophyll fluorescence intensity and white bars give the scale (20 µm) for populations in low (green) and high (orange) salinities. (b) Morphological variation in cells after 10 days in low (green) and high (orange) salinities for populations that have evolved under predictable (upper graph) vs. unpredictable (lower graph) environmental fluctuations. (c). Evolution of the degree of plasticity at day 10. Standard error based on 1000 bootstraps is also plotted for each dot, but not visible. Red line is the regression slope, and the grey lines show 1000 regression slopes calculated on bootstrapped samples.

**Figure 3:**
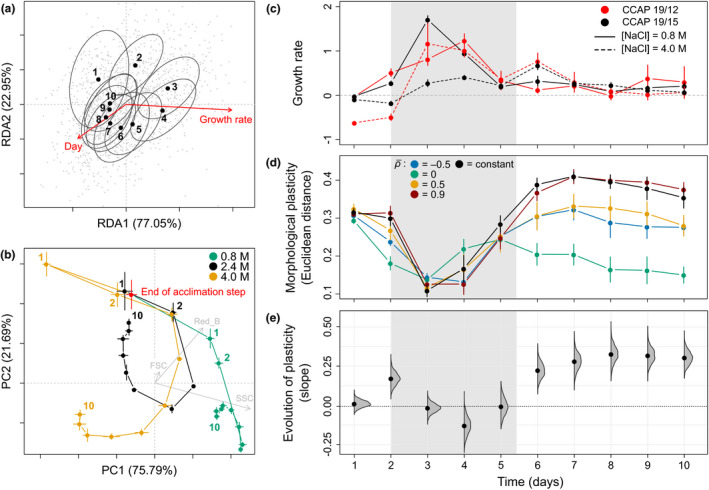
Evolution of plasticity along the ontogenic trajectory. (a) The ontogenic trajectory of cell morphology for an isogenic population transferred to fresh medium with unchanged salinity is shown as centroids (dots) and covariance matrices (ellipses) for each day. Arrows indicate the effects of population growth rate and day during the assay. (b) Trajectories of four isogenic populations from the same evolved line in three different salinities. PC loadings of flow cytometry measurements (grey arrows) were rescaled by dividing them by 20 to facilitate graphical representation. (c) Per capita population growth rate per day, across all evolved lines. (d) Temporal variation in the degree of phenotypic plasticity of the four treatments of expected long‐term temporal autocorrelation ($\bar{\rho }$), and populations that evolved in constant salinity (black). (e) Evolution of plasticity along the ontogeny. The mean slope of the regression of the degree of plasticity (Euclidian distance between environments) against environmental predictability (ρ^2^) is shown for each day post‐transfer to a new salinity. Density plots represent slopes estimated on 1000 bootstrapped samples of the raw data (same as grey lines in Fig. 2c for day 10). For (c) to (e), the grey dashing delimits the phase with highest growth rate. Means with their standard errors are represented as dots and error bars respectively.

We first investigated the plasticity of cells that had approached phenotypic equilibrium, 10 days after transfer to a new salinity. Comparison of samples from low vs. high salinities revealed a clear signal of plasticity, with a significant effect of salt concentration on cell morphology (Table 1). Cells from high salinity were smaller and contained less chlorophyll than cells from low salinity (Fig. 2a & Fig. S2a). However, this plasticity was not identical in all experimental lines: lines that evolved in different environmental predictabilities differed in their plastic responses to salinity (Fig. 2b; significant *salinity* × ρ^2^ interaction in Table 1). The magnitude of plasticity, as quantified by the Euclidian distance of phenotypic change between low and high salinities (length of black segments in Fig. 2b, & Fig. S2b), was positively correlated to the environmental predictability during experimental evolution, indicating that lines that had experienced less predictable environments during the experimental evolution phase evolved reduced phenotypic plasticity (linear regression: *R* ^2^ = 0.621, *P* < 0.001; Fig. 2c). This result was replicated over the two ancestral strains (Fig. 2c) despite their differences on other features such as their population dynamics (Fig. 3 & Fig. S3), and also held for isogenic populations (Fig. S4a). The latter confirmed that the observed morphological differences between salinities were the result of phenotypic plasticity, rather than rapid selection of salinity‐specific genotypes over the assay phase (since isogenic populations display no genetic variation).

**Table 1:** Partitioning of cell morphological variation

	Factors	d.f.	Variance (×10^−3^)	*F*	*R* ^2^
Evolution in fluctuating salinity	Strain	1	0.926	2069.386	0.010***
	Growth Rate	1	2.402	5368.318	0.026***
	Day	9	10.456	2596.641	0.113***
	Salinity	2	19.025	21261.528	0.205***
	Predictability (ρ^2^)	1	2.084	4657.919	0.022***
	Salinity × ρ^2^	2	1.377	1539.408	0.015***
	Day × Salinity	18	3.770	468.150	0.041***
	Day × ρ^2^	9	0.186	46.164	0.002***
	Day × Salinity × ρ^2^	18	0.346	42.931	0.004***
	Residual	116 636	52.184		0.563
Evolution in constant salinity	Strain	1	3.790	1048.620	0.042***
	Growth Rate	1	1.856	513.564	0.021***
	Day	9	15.808	485.962	0.176***
	Salinity	1	22.000	6087.119	0.245***
	Day × Salinity	9	3.226	99.183	0.036***
	Residual	11 880	42.938		0.479

The effect of all explanatory factors and their interactions on multivariate patterns of cellular variation are quantified by their *R* ^2^ (proportion of total variation explained), and the significance of each *R* ^2^ was tested by ANOVA‐like permutation tests using 999 randomisations of the data (Legendre and Legendre, 1998). Salinity refers to the salinity of the culture on the day it was measured during the plasticity assay.

*** *P *< 0.001.

We also maintained four lines at constant salinity ([NaCl] = 2.4 M) throughout the experiment, as controls for the influence of environmental fluctuations. Lines that evolved in constant environments (right panel in Fig. 2c and Fig. S4a) had a similar degree of plasticity as lines evolved under high predictability values, from *ρ^2^* = 0.6 to ρ^2^ = 0.8 (*P* = 0.295, such that the hypothesis of identical degree of plasticity cannot be rejected). This suggests that unpredictable environments exerted a stronger selective pressure against plasticity (Gavrilets and Scheiner, 1993; Lande, 2009) than any putative cost associated to the maintenance of plasticity in a constant environment (DeWitt *et al*., 1998). Interestingly, we found similar degrees of plasticity between populations that evolved in treatments with autocorrelations $\bar{\rho }$ = −0.5 and $\bar{\rho }$ = 0.5 (*P* = 0.867), which have the same expected predictability of changes (ρ^2^ = 0.25), but different magnitude of transitions upon each transfer (Fig. S5).

### Ontogeny of evolved plasticity

We then turned to the entire morphological trajectory over 10 days, to investigate how the evolution of plasticity unfolds along developmental time scales. When cells were transferred at low density (*c*. 2 × 10^4^ cells.ml^−1^) to fresh medium with unchanged salinity, their morphology followed a loop‐shaped trajectory over 10 days, with initial and final phenotypes markedly differing from phenotypes at intermediate times (Fig. 3a). These changes concerned cell size and granularity for the first days (up to days 2‐3: bigger cells when reaching the exponential phase), followed by changes in chlorophyll content (days 3‐4 to 10: decreasing chlorophyll content in the stationary phase) (Fig. 3a and b & Fig. S1a). As this temporal trend in morphology was also found for isogenic populations with effectively no opportunity for natural selection (Fig. 3a and b), it does not reflect genetic changes in the population. Instead, these changes can be described as ontogenic, in line with the extended definition of ontogeny/development as a sequence of cellular states (including through cell division), which applies across unicellular and multicellular organisms (Gilbert, 2000). Part of this ontogenic morphological variation was explained by population growth rate (Table 1), an aggregate population‐level outcome of life‐history traits of individual cells (division and death rate), which is typically used as an indicator of physiological state in microbiology (Maharjan *et al*., 2013). Focusing on populations maintained in the same salinity as during acclimation ([NaCl] = 2.4 M), we detected a clear effect of growth rate (*R* ^2^ = 0.046, *P* < 0.001) on morphological variation (red arrow in Fig. 3a; same effect for both ancestral strains, *growth rate × strain* interaction: *P* = 0.504), independent from salinity changes. However, the population growth rate was not sufficient to explain the ontogenic trajectory, and there remained a significant marginal effect of the time spent in the new environment (*R* ^2^ = 0.031, *P* < 0.001).

Ontogenic trajectories also differed between salinities. Upon transfer to a new salinity, morphologies in the hypo‐ vs. hyper‐osmotic environments first rapidly diverged in opposite directions from the acclimated morphology, 4 h after salinity change (day 1 in green and yellow vs. red dot; Fig. 3b), before converging during the exponential phase, and finally diverging again to salinity‐specific morphologies over the stationary phase (Fig. 3b & Fig. S2a). This is consistent with known responses of *D*.* salina* to salinity change, which first involve an immediate – sometimes drastic – change in cell volume caused by water intake/loss, followed by slower accumulation of salt‐induced proteins and metabolites (notably via changes in gene expression Chen and Jiang, 2009; Fang *et al*., 2017), which restore cell shape and eventually lead to long‐term changes in cell content such as lipid, carotene and glycerol accumulation (Ben‐Amotz *et al*., 2009). As a result of these differences in morphological trajectories between salinities, the plasticity of cell morphology was temporally variable (significant *day × salinity* interaction; Table 1). Plastic differences in morphology also followed a loop over 10 days, where initial and final differences diverged from those at intermediate times (Fig. S2b). The degree of plasticity was highest at low population growth rates characteristic of the lag and stationary phases, and lowest during the exponential phase (significant *salinity × growth rate* interaction, Table 1, Fig. 3c and e and Fig. S2).

Evolution of plasticity in response to environmental predictability had different impacts at distinct stages of the ontogenic trajectory. We found a positive relationship between the level of plasticity and environmental predictability during phases of slow population growth (at day 2, and from days 6 to 10; Fig. 3c and e), but no relationship at day 1 and during the exponential phase (days 3 to 4; Fig. 3c and e; similar results were observed for isogenic populations, Fig. S4b). That there was little evolution of plasticity during the exponential phase is consistent with the finding that morphological plasticity is generally lowest in this phase (Fig. 3c and d). In contrast, plasticity is high shortly after salinity transfer (day 1, Fig. 3d), but mostly because of passive, reflex responses to osmotic stress, which are certainly less prone to evolution than longer term physiological responses involving specific gene expression and production of metabolites and chlorophyll.

## DISCUSSION

We have shown that phenotypic plasticity, a major component of phenotypic change in the wild (Scheiner, 1993; Schlichting and Pigliucci, 1998; West‐Eberhard, 2003; Pelletier *et al*., 2007; Ozgul *et al*., 2010; Ellner *et al*., 2011), and a key mechanism for population persistence in a changing environment (Chevin *et al*., 2010; Reed *et al*., 2010; Vedder *et al*., 2013; Chevin *et al*., 2013b; Ashander *et al*., 2016; Phillimore *et al*., 2016), can evolve experimentally in response to environmental predictability, in the direction predicted by theory (Gavrilets and Scheiner, 1993; de Jong, 1999; Lande, 2009; Botero *et al*., 2015; Tufto, 2015). This plasticity concerned morphological traits that were previously described as being involved in salinity tolerance in *Dunaliella salina* (Azachi *et al*., 2002; Oren, 2005; Ben‐Amotz *et al*., 2009), a species for which we have shown that plastic responses to past environments can largely drive population dynamics and extinction risk in a randomly fluctuating environment (Rescan *et al*., 2020).

Interestingly, we experimentally confirmed the prediction that lower plasticity evolves in less predictable stochastic environments (Gavrilets and Scheiner, 1993; de Jong, 1999; Lande, 2009; Botero *et al*., 2015; Tufto, 2015), even though our study system and experimental design differ to some extent from underlying assumptions of the theory. In particular, most of the theory assumes that the environment changes every generation, and that the trait is fixed once during development and later exposed to selection (but see e.g. Lande (2014) or Ratikainen and Kokko (2019) for more complex theory on evolution of reversible plasticity). In contrast, we here changed the environment every few generations (3 to 4 days), and the traits exhibited transient plastic changes for several days (and thus generations) after exposure to a new salinity (Figure 2). (Note, however, that generations have a somewhat different meaning for unicellular organisms that reproduce mostly clonally, such that many phenotypic traits are directly transmitted to daughter cells upon division). That a key theoretical prediction about the evolution of plasticity is verified empirically in our experiment implies that even simple theory can capture essential biological features that are relevant in more complex experimental systems, namely delays between exposure to the environment, trait development and selection.

Contrary to common practice in experimental evolution with microbes (Elena and Lenski, 2003; Buckling *et al*., 2009), we have assayed evolved changes by measuring multiple individual traits (rather than aggregate population traits) across environments and over time. This approach tending towards phenomics allowed us to describe an ontogenic sequence of cell morphology, consistent with osmotic response mechanisms operating at different time scales. These responses ranged from immediate physical change in cell shape (passive plasticity) occurring within the first few seconds/minutes, to long‐term physiological regulations involving changes in gene expression (active plasticity), which usually start within 12–24 h (Chen and Jiang, 2009; Fang *et al*., 2017). These consecutive morphological states of cells, which we here described as an ontogenic sequence (Gilbert, 2000), can also be interpreted as alternative phenotypes favored at different population densities [*r* vs. *K*‐selection (MacArthur, 1962; Sæther *et al*., 2016)], or plastic responses to environmental variables other than salinity that change along time in a batch culture (e.g. resource abundance and quality) (Collot *et al*., 2018).

Evolution of plasticity in response to environmental predictability was essentially restricted to physiological states associated with phases of slow – or even null – population growth in our experiment. Population growth status, indicative of the physiological state of individual cells, is known to be associated with multiple phenotypic traits of micro‐organisms (Maharjan *et al*., 2013). The lack of plasticity during the exponential phase is likely to result from a particular morphology associated with rapid cell division, which masks the influence of salinity. Nevertheless, the morphological changes expressed as late responses to osmotic stress (day 5–10) probably result from internal cellular changes that were initiated as soon as the start of active plasticity (day 2), as suggested by the fact that we already observed the evolution of reduced plasticity in less predictable environments at the beginning of the ontogenic sequence of cell morphology.

Our results demonstrate that long‐term experimental evolution under complex, ecologically realistic patterns of environmental variation, coupled with intense high‐throughput phenotyping at the individual level, allows testing fundamental predictions in evolutionary ecology that are barely approachable in natural settings. Our finding that phenotypic plasticity can evolve in response to environmental predictability proves important for the prospects for population persistence in the face of global warming, and other anthropogenic changes. Indeed these changes consist not only of trends in mean environments, but also alterations in the magnitude and predictability of natural environmental fluctuations (Wigley *et al*., 1998; Boer, 2009). Theoretical work has made it clear that phenotypic plasticity can strongly influence extinction risk in response to changing environmental predictability (Reed *et al*., 2010; Chevin *et al*., 2013a; Botero *et al*., 2015; Ashander *et al*., 2016), which was recently confirmed empirically using laboratory experiments (Proulx *et al*., 2019; Rescan *et al*., 2020). In particular, this theory has shown that evolution of lower phenotypic plasticity can reduce extinction risk under reduced environmental predictability, by decreasing the magnitude of mismatches between the population mean phenotype and the optimum phenotype determined by the environment (Chevin *et al*., 2013a; Ashander *et al*., 2016). Our demonstration that such evolution of reduced plasticity can indeed occur over a few hundred generations indicates that this may be an important mechanism by which species could persist under climate change.

## COMPETING INTEREST

Authors declare no competing interests.

## AUTHORSHIP

L‐MC and MR designed, and MR, DG and CL performed the experimental evolution experiment. CL designed and performed the plasticity experiments, analysed the data, prepared figures and table and wrote the first drafts of the paper with contributions from L‐MC. All authors reviewed and approved the final draft of the paper.

### Peer Review

The peer review history for this article is available at https://publons.com/publon/10.1111/ele.13598.

## Supporting information

Supplementary Material

## Data Availability

Data that support the findings of this study are archived in Figshare Repository: https://doi.org/10.6084/m9.figshare.12770018.
