# Supplementary material for: Reduced phenotypic plasticity evolves in less predictable environments
Source: Ecol Lett. 2020 Aug 31;23(11):1664–72. doi: 10.1111/ele.13598 (PMC7754491; doi:10.1111/ele.13598)
Supplement: Supplementary file 1 — Supplementary Material [file ELE-23-1664-s001.docx]

**Reduced phenotypic plasticity evolves in less predictable environments**

Christelle Leung^1^, Marie Rescan^1^, Daphné Grulois^1^ & Luis-Miguel Chevin^1^

^1^ CEFE, Université de Montpellier, CNRS, EPHE, IRD, Université Paul Valéry Montpellier 3, Montpellier, France.

*Correspondence:* [*christelle.leung@umontreal.ca*](mailto:christelle.leung@umontreal.ca)

Supplementary information include:

- Supplementary Material 1: Comparison of flow cytometry *vs* confocal microscopy methods to assess cells morphology.
- Figure S1: Comparison of flow cytometry vs confocal microscopy methods to assess cells morphology.
- Figure S2. Ontogenetic trajectories and dynamics of plasticity over time.
- Figure S3. Population dynamics in low and high salinities.
- Figure S4. Analyses of isogenic populations.
- Figure S5. Influence of the magnitude of environmental transitions for a given environmental predictability.

Supplementary Material 1: Comparison of flow cytometry *vs* confocal microscopy methods to assess cells morphology

We carried out microscopy images analyses on a small subset of populations, to confirm the accuracy of flow cytometry for assessing cell morphology. As for the main plasticity assay, we first acclimatized four randomly chosen evolved populations to the mean salinity ([NaCl] = 2.4 M) during 10 days. We then transferred each of them to low ([NaCl] = 0.8 M) or high ([NaCl] = 4.0 M) salinity for 10 others days. We finally measured cell morphology using flow cytometry and dragonfly confocal microscopy.

Dragonfly confocal microscopy: We sampled ~40µL of cultures from each condition (*n* = 8, for 4 populations in 2 salinities), and scanned them on a Dragonfly (COM4) spinning disk confocal microscope with 2 cameras EMCCD iXon888 Life (Oxford Instrument - Andor). A total of 100 images was taken under both transmission and confocal fluorescence modes, to assess cells shape and chlorophyll content, respectively. Wavelengths excitation and emission were at 488 nm and 700/775 nm, respectively, for confocal fluorescence.

We then used the automatic image analysis software Fiji (Schindelin *et al.* 2012) to characterize cells morphology. First, we used the function *Analyze Particles* to isolate cells in each picture, and manually validate each detected particle as an actual *D. salina* cell. For each particle, we then recorded morphological descriptors including the area and aspect ratio (AR: major/minor axis), the average gray - sum of the gray values of all the pixels in the selection divided by the number of pixels (Grey_Int and Grey_sd for mean and standard error respectively) - for the transmission mode, and fluorescence intensity (Fluo_Int and Fluo_sd for mean and standard error respectively) within the selection.

Flow cytometry: We passed a 150 µL sample of each condition through flow cytometry, using the same method as described in the main text. We then randomly selected 30 events characterized as alive *Dunaliella salina* cells per condition, to keep a balanced sampled size with the microscopy dataset. We then used FSC, SSC and Red-B values, followed by light signal correction using Guava^®^ EasyCheck™ kit calibrating beads, to assess cells morphology.

Statistical analyses: We tested the concordance of the morphospaces obtained by using the two methods, as proposed by Wang *et al.* (2010). We first carried out independent principal component analyses of morphology for flow cytometry and microscopy. We thereafter extracted the centroids on PCA plot for each group of cells, characterized by their population and salinity. Finally, we aligned the centroids’ PCA coordinates of flow cytometry morphospace with the microscopy ones using a Procrustean superimposition approach (Gower 1975; Legendre & Legendre 1998). The degree of concordance between the ordinations was assessed with a correlation-like statistic *r-*PROTEST(Jackson 1995), derived from the symmetric sum of squares, where 1 and 0 denote a perfect vs totally absent concordance, respectively. The significance of this correlation was tested by permutation, using 999 randomizations against the null hypothesis that there is no concordance between the two ordinations (Jackson 1995).

*Supplementary Information References:*

Gower, J.C. (1975). Generalized Procrustes analysis. *Psychometrika*, 40, 33-51.

Jackson, D.A. (1995). PROTEST: a PROcrustean Randomization TEST of community environment concordance. *Ecoscience*, 2, 297-303.

Legendre, P. & Legendre, L. (1998). *Numerical ecology*. 2nd English edition edn. Elsevier Science, Amsterdam.

Schindelin, J., Arganda-Carreras, I., Frise, E., Kaynig, V., Longair, M., Pietzsch, T. *et al.* (2012). Fiji: an open-source platform for biological-image analysis. *Nat. Methods*, 9, 676-682.

Wang, C., Szpiech, Z.A., Degnan, J.H., Jakobsson, M., Pemberton, T.J., Hardy, J.A. *et al.* (2010). Comparing spatial maps of human population-genetic variation using Procrustes analysis. *Statistical Applications in Genetics and Molecular Biology*, 9.


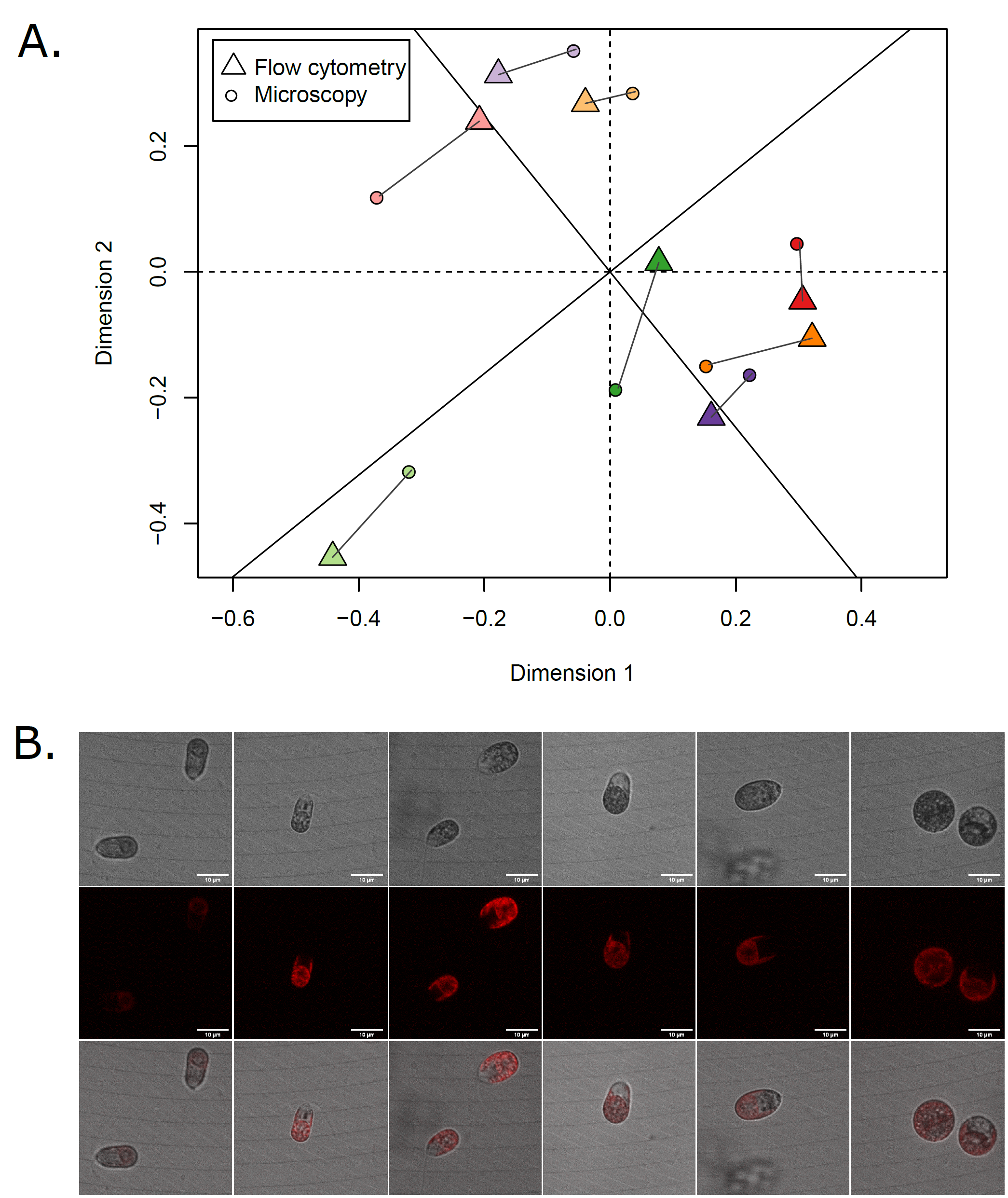


**Figure S1. Comparison of flow cytometry vs confocal microscopy methods to assess cells morphology.** A. Procrustes errors between PCAs performed on flow cytometry (triangle) and confocal microscopy (circle) parameters. Each color represent the centroids of a given population, with lighter vs darker colors for low (0.8 M) vs high (4.0 M) salinity, respectively. B. Example of cell morphology. Images were taken using a dragonfly confocal microscope, under the transmission (first line) and confocal in fluorescence (second line) acquisitions respectively, and a composite of both acquisitions (third line, similar to Fig. 2B in the main text). White scale bar represents 10µm.


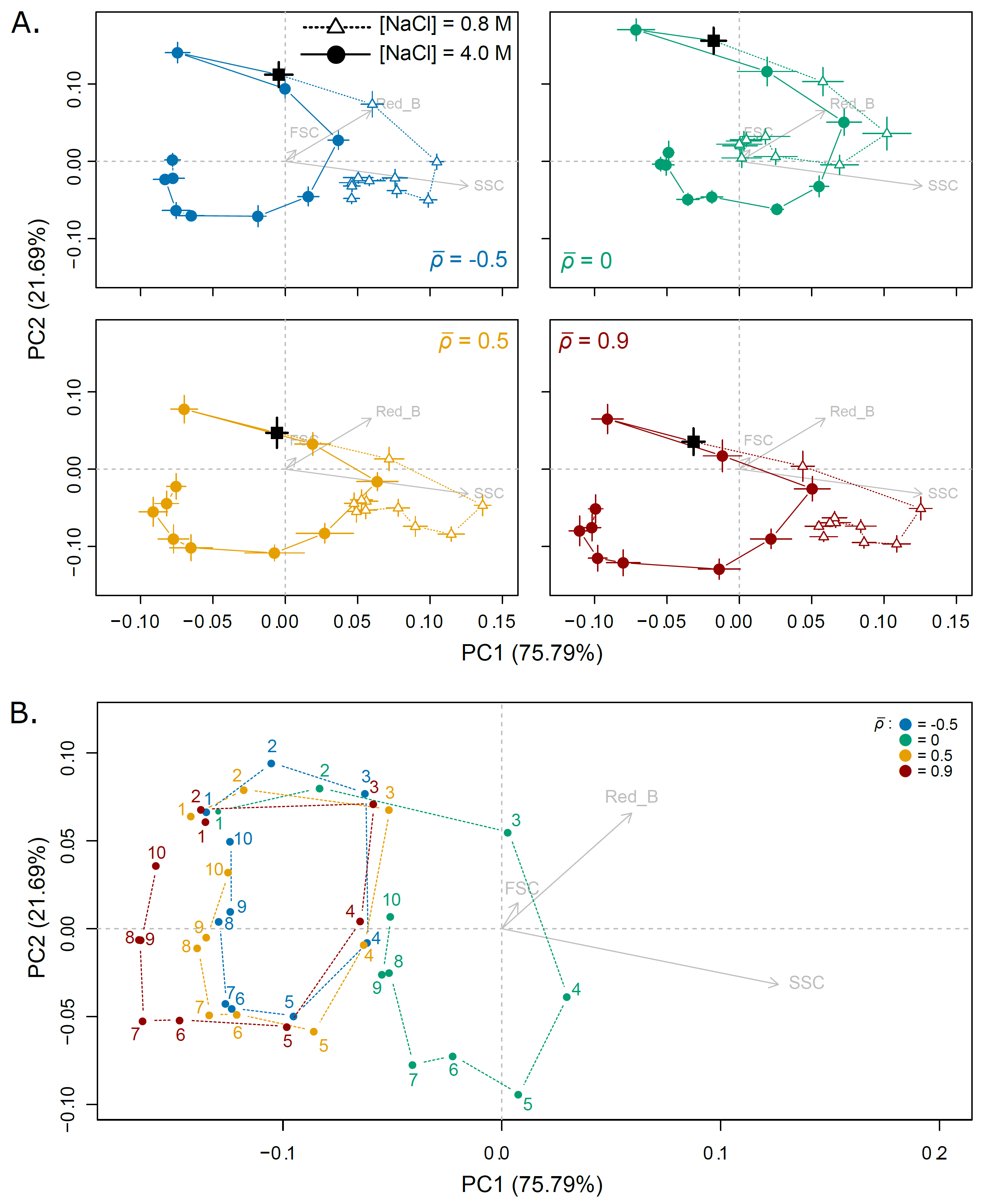


**Figure S2. Ontogenetic trajectories and dynamics of plasticity over time.** A. Morphological trajectories in two salinities for each autocorrelation treatment ($\bar{\rho}$). Mean cell morphologies with their standard errors, per day and salinity, are calculated by averaging over lines belonging to the same targeted $\bar{\rho}$, and represented along the two first PCA axes. Vectors of PC loadings of response variables (FSC, SSC and Red_B grey arrows) were divided by 20 to facilitate graphical representation. Black squares represent cell morphology at the end of acclimation step. B. Trajectories of plasticity over time. Morphological differences between salinities for each treatments were computed as vector at high salinity *minus* the one at low salinity, for each day (number). Coordinates 0,0 in this morphological space means the absence of plasticity.


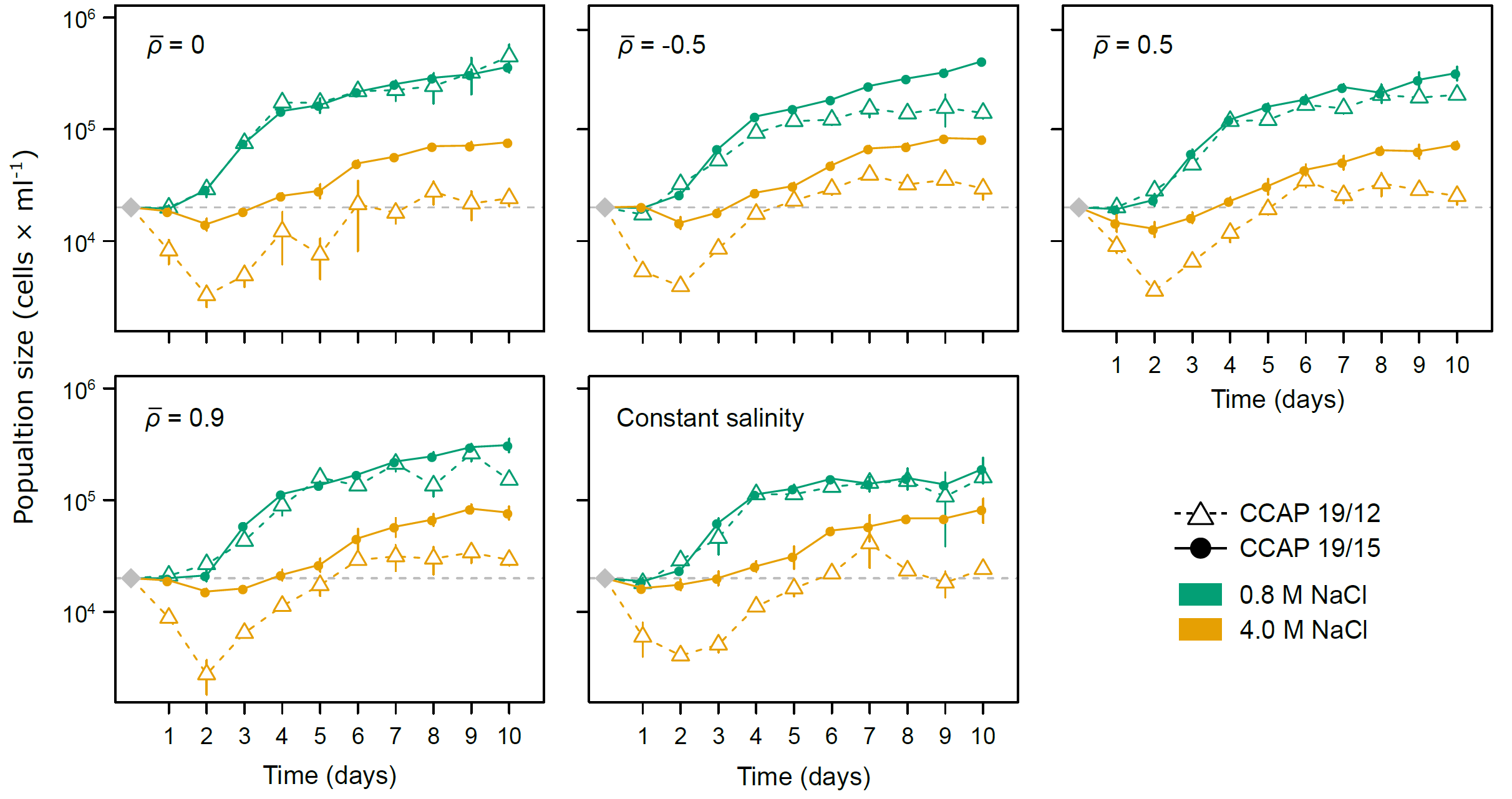


**Figure S3. Population dynamics in low and high salinities.** For each evolutionary treatment (temporal autocorrelation ($\bar{\rho}$) or constant salinity at [NaCl] = 2.4M), mean population size (cells × ml^-1^) with their standard errors, per day and salinity, were calculated by averaging over lines belonging to the same strain. Grey diamond is the expected initial population size (2 × 10^4^ cells × ml^-1^) inoculated in the new salinity at the beginning of the plasticity assay, and day 1 was measure 4h after this salinity change.


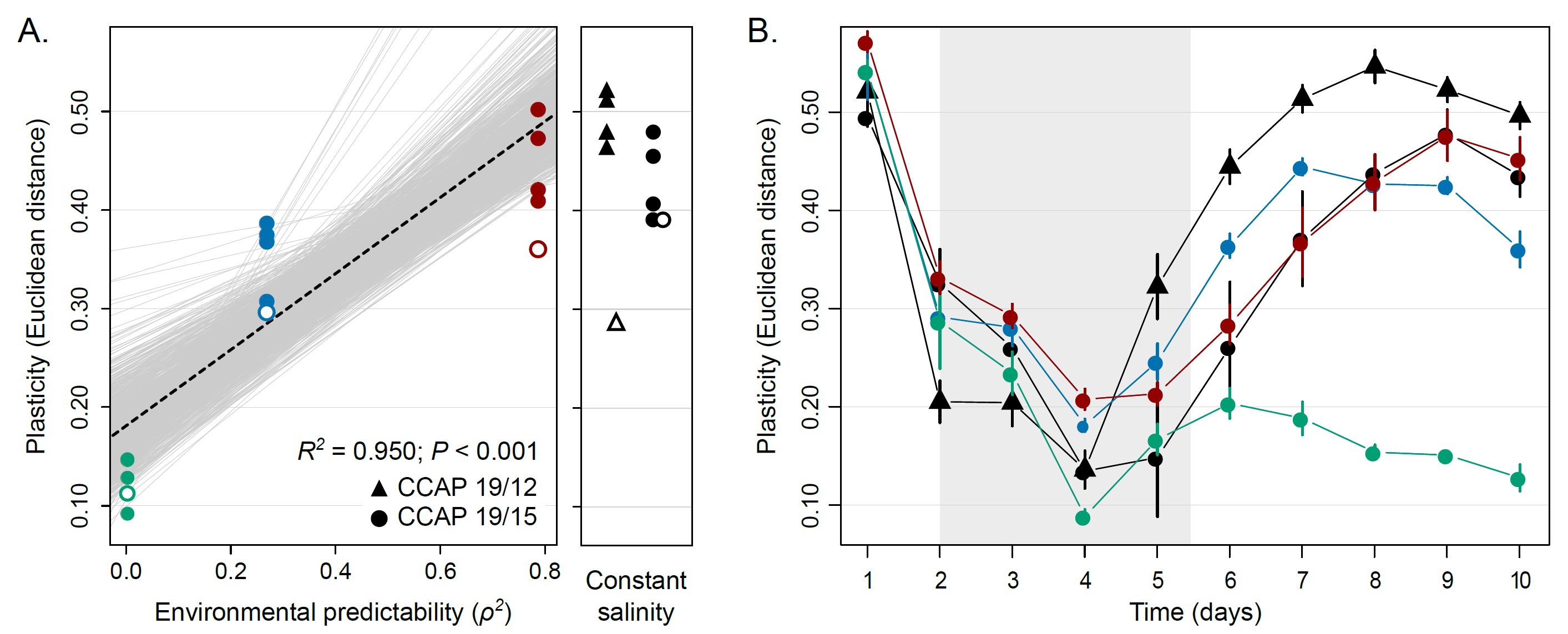


**Figure S4. Analyses of isogenic populations.** A. Evolution of plasticity at day 10. Isogenic populations were founded from a single cell from three different evolved lines under *ρ* = -0.022 (green), *ρ* = -0.522 (blue) and *ρ* = 0.889 (red) and two strain (CCAP 19/12 as triangle and CCAP 19/15 as circle) from constant environment. The degree of plasticity of populations, measured as the Euclidian distance of mean cell morphology between low and high salinities, is plotted against the predictability *ρ^2^* of environmental fluctuations of these populations that have experienced during experimental evolution. Standard error based on 1000 bootstraps are also plotted, but not visible. The regression slope is represented as black dashed line, and the grey lines encompass 1000 regression slopes calculated for each bootstrap. The panel on the right shows plasticity in populations that evolved under constant salinity, corresponding to the mean of fluctuating treatments. Variation partitioning revealed significant morphological differences among isogenic populations founded from a given evolved lines (*R^2^* = 0.009; *P <* 0.001), but those remained smaller than morphological variation among different evolved lines (*R^2^* = 0.051; *P <* 0.001), when controlling for the effect of salinity, day, and their interaction on the total variation. Filled and open symbols represent isogenic populations and the evolved lines they come from, respectively. B. Temporal variation in the degree of phenotypic plasticity. For each population, Euclidean distances were computed between mean cell morphologies in [NaCl] = 0.8M vs 4.0M. Means and standard errors were assessed from 4 isogenic populations for each evolved lines.


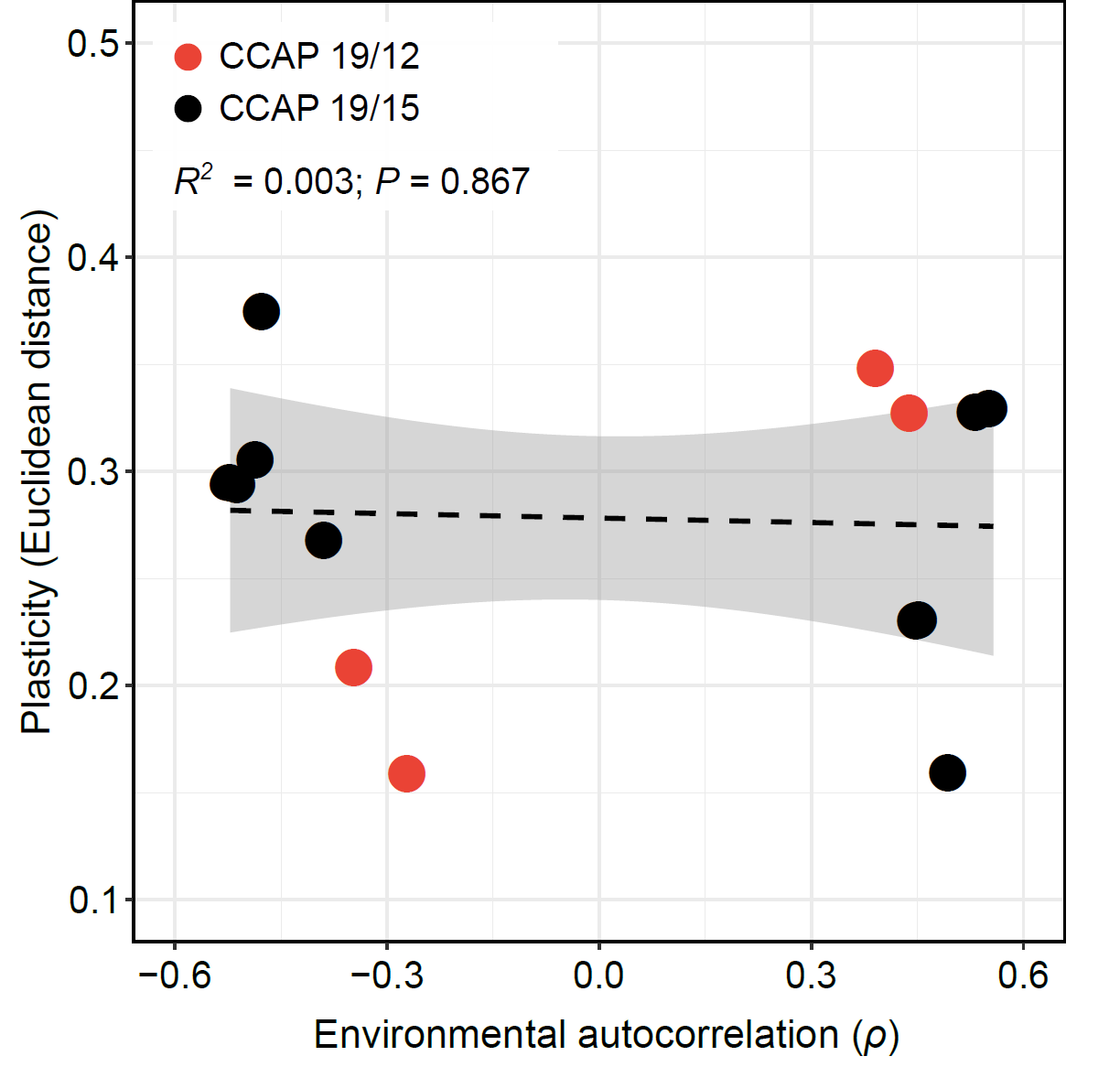


**Figure S5. Influence of the magnitude of environmental transitions for a given environmental predictability.** The degree of plasticity of populations, measured as the mean Euclidean distance of cell morphology after 10 days in low vs high salinities, is plotted against the environmental autocorrelation ($\bar{\rho}$). Negative and positive autocorrelation displayed the same environmental predictability ($\bar{\rho}^{2}$*=* 0.25), but differed in the intensity of environmental changes between two successive transfers. The regression slope is also represented as black dashed line, and the shaded region shows the 95% CI of this regression.
